# Dominant subtype switch in avian influenza viruses during 2016–2019 in China

**DOI:** 10.1038/s41467-020-19671-3

**Published:** 2020-11-20

**Authors:** Yuhai Bi, Juan Li, Shanqin Li, Guanghua Fu, Tao Jin, Cheng Zhang, Yongchun Yang, Zhenghai Ma, Wenxia Tian, Jida Li, Shuqi Xiao, Liqiang Li, Renfu Yin, Yi Zhang, Lixin Wang, Yantao Qin, Zhongzi Yao, Fanyu Meng, Dongfang Hu, Delong Li, Gary Wong, Fei Liu, Na Lv, Liang Wang, Lifeng Fu, Yang Yang, Yun Peng, Jinmin Ma, Kirill Sharshov, Alexander Shestopalov, Marina Gulyaeva, George F. Gao, Jianjun Chen, Yi Shi, William J. Liu, Dong Chu, Yu Huang, Yingxia Liu, Lei Liu, Wenjun Liu, Quanjiao Chen, Weifeng Shi

**Affiliations:** 1 CAS Key Laboratory of Pathogenic Microbiology and Immunology, Institute of Microbiology, Center for Influenza Research and Early-warning (CASCIRE), CAS-TWAS Center of Excellence for Emerging Infectious Diseases (CEEID, Chinese Academy of Sciences, 100101 Beijing, China; 2 University of Chinese Academy of Sciences, 101408 Beijing, China; 3 Shenzhen Key Laboratory of Pathogen and Immunity, Guangdong Key Laboratory for Diagnosis and Treatment of Emerging Infectious Diseases, State Key Discipline of Infectious Disease, Second Hospital Affiliated to Southern University of Science and Technology, Shenzhen Third People’s Hospital, 518112 Shenzhen, China; 4 Key Laboratory of Etiology and Epidemiology of Emerging Infectious Diseases in Universities of Shandong, Shandong First Medical University & Shandong Academy of Medical Sciences, 271016 Taian, China; 5 Institute of Animal Husbandry and Veterinary Medicine, Fujian Academy of Agricultural Sciences, 350013 Fuzhou, China; 6 China National Genebank-Shenzhen, BGI-Shenzhen, 518083 Shenzhen, China; 7 College of Life Science and Technology, Xinjiang University, 830046 Urumchi, China; 8 Zhejiang Provincial Engineering Laboratory for Animal Health Inspection & Internet Technology, College of Animal Science and Technology & College of Veterinary Medicine of Zhejiang A&F University, 311300 Hangzhou, China; 9 College of Animal Science and Veterinary Medicine, Shanxi Agricultural University, 030801 Taigu, China; 10 Institute of Zoonosis, College of Public Hygiene, Zunyi Medical University, 563003 Zunyi, China; 11 College of Veterinary Medicine, Northwest A&F University, 712100 Yangling, Shaanxi China; 12 Department of Veterinary Preventive Medicine, College of Veterinary Medicine, Jilin University, 130062 Jilin, China; 13 School of Basic Medicine and Life Science, Hainan Medical University, 571101 Haikou, China; 14 Diqing Tibetan Autonomous Prefecture Centers for Disease Control and Prevention, 674400 Shangri-la, China; 15 CAS Key Laboratory of Special Pathogens and Biosafety, Wuhan Institute of Virology, Center for Biosafety Mega-Science, CASCIRE, Chinese Academy of Sciences, 430071 Wuhan, China; 16 College of Animal Science and Technology, Henan Institute of Science and Technology, 453003 Xinxiang, China; 17 College of Animal Science, Southwest University, 402460 Chongqing, China; 18 Institut Pasteur of Shanghai, Chinese Academy of Sciences, 200031 Shanghai, China; 19 Département de microbiologie-infectiologie et d’immunologie, Université Laval, Québec City, G1V 0A6 Canada; 20 Federal Research Center of Fundamental and Translational Medicine, Federal State Budget Scientific Institution, Siberian Branch of Russian Academy of Sciences, Novosibirsk State University, Novosibirsk, Russia 630090; 21 National Institute for Viral Disease Control and Prevention, Chinese Center for Disease Control and Prevention (China CDC), 102206 Beijing, China; 22 General Station for Surveillance of Wildlife-borne Infectious Diseases, State Forestry and Grassland Administration, 110034 Shenyang, Liaoning Province PR China; 23 School of Public Health, Shandong First Medical University & Shandong Academy of Medical Sciences, 271000 Taian, China

**Keywords:** Phylogenetics, Genome, Influenza virus, Epidemiology

## Abstract

We have surveyed avian influenza virus (AIV) genomes from live poultry markets within China since 2014. Here we present a total of 16,091 samples that were collected from May 2016 to February 2019 in 23 provinces and municipalities in China. We identify 2048 AIV-positive samples and perform next generation sequencing. AIV-positive rates (12.73%) from samples had decreased substantially since 2016, compared to that during 2014–2016 (26.90%). Additionally, H9N2 has replaced H5N6 and H7N9 as the dominant AIV subtype in both chickens and ducks. Notably, novel reassortants and variants continually emerged and disseminated in avian populations, including H7N3, H9N9, H9N6 and H5N6 variants. Importantly, almost all of the H9 AIVs and many H7N9 and H6N2 strains prefer human-type receptors, posing an increased risk for human infections. In summary, our nation-wide surveillance highlights substantial changes in the circulation of AIVs since 2016, which greatly impacts the prevention and control of AIVs in China and worldwide.

## Abstract (web summary)

In this study, the authors present a genomic surveillance of avian influenza genomes sampled from live poultry markets in China. They report that a number of variants have emerged since 2016 that pose an increased risk to humans. They highlight the importance of continuous genome surveillance of circulating influenza strains.

## Introduction

Avian influenza viruses (AIVs) of various subtypes, e.g. H9N2 low pathogenic AIV (LPAIV) and H5Ny highly pathogenic AIV (HPAIV), have been circulating throughout China and elsewhere in the world^1,2^, causing huge economic losses. In particular, these AIVs were shown to be able to infect humans^3–5^. As of 24 June 2019, at least 861 human cases with H5N1 infections have been reported globally^6^. Although there were only 50 reported human cases since the first human infection with H9N2 AIVs in 1998^4,5^, a seroprevalence rate of 11.20% against H9N2 AIVs among healthy occupational workers was observed in several provinces of China during 2014–16, which is substantially higher than those against H7N9, H5N1, H5N6, H6N1, and H6N6 AIVs^7^, implying that H9 AIV has a higher infectivity to humans than other AIVs and can cause transient human infections.

Worryingly, a number of novel reassortant subtypes including H7N9, H6N1, H10N8, H5N6, and H7N4 were reported to infect humans^5,8–12^. In particular, evidence has shown that H9N2 contributed to the emergence and evolution of these novel human-infecting AIVs (e.g. H7N9, H10N8, and H5N6) and that poultry carrying H9N2 in live poultry markets (LPMs) may act as the genetic incubator for creating novel reassortant AIVs^13–16^. This highlights the importance of continuous surveillance of AIVs in LPMs.

In our previous report, we have established the Center for Influenza Research and Early-warning, Chinese Academy of Sciences (CASCIRE) surveillance network and performed monitoring studies for AIVs during 2014–16 in LPMs in China^13,17^. We found that while H9N2 was the dominant subtype in northern China, H5N6 has replaced H5N1 as a dominant AIV subtype in southern China. Importantly, H5N6 seems to be more virulent than H5N1 in humans based on the clinical data and case fatality rates (CFRs) (H5N6: ~69.60% and H5N1: ~52.50%)^18^, even though only 23 human H5N6 cases have been reported thus far^5^. In addition, H9N2 was primarily isolated from chickens, while H5N6 was mainly isolated from ducks^13^. Almost simultaneously, H5N8 HPAIV spread globally and caused outbreaks in migratory birds in Asia, Europe, and North America^1,5,19^. Furthermore, H7N9 HPAIV emerged in 2016^20,21^ with a higher CFR compared to H7N9 LPAIV in humans^22^. Remarkably, the number of human H7N9 cases reported in the 2016–17 influenza season alone approximately equalled all of the previously recorded cases during 2013–16^5,23^.

In the present study, we continued our previous work from 2014–16 and report the results of nation-wide AIV surveillance in China during 2016–19. Our results show that the AIV positive rate at LPMs substantially decreased compared with that during 2014–16. H9N2 has become the dominant subtype both in chickens and ducks across China. In contrast, H7N9 has almost disappeared in 2018. Furthermore, H7N3 reassortants, H5N6 HPAIV variants, as well as H9N9 and H9N6 LPAIV reassortants have emerged, warranting constant monitoring.

## Results

### AIV positive rates significantly decreased during 2016–19

**Fig. 1:**
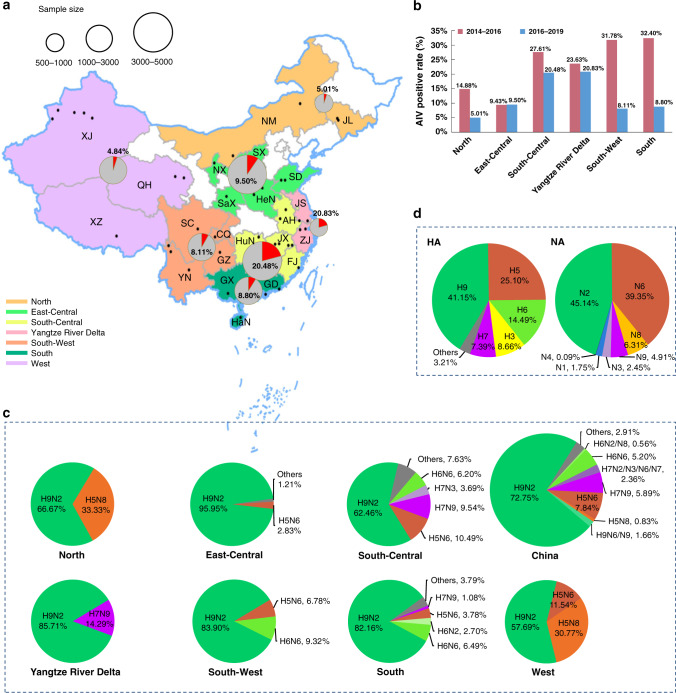
The distribution of sampling sites and avian influenza viruses (AIVs) in LPMs across China. **a** Map of the AIV sampling sites and isolation rates in LPMs. AIV surveillance sites in 37 cities (indicated by black dots) of 23 provinces or municipalities or minority autonomous regions in China are divided into seven different regions: North (Inner Mongolia, NM and Jilin, JL; orange), East-Central (Shanxi, SX; Ningxia, NX; Shandong, SD; Shaanxi, SaX and Henan, HeN; light green), South-Central (Anhui, AH; Hunan, HuN; Jiangxi, JX and Fujian, FJ; yellow), Yangtze River Delta (Jiangsu, JS and Zhejiang, ZJ; pink), South-West (Sichuan, SC; Chongqing, CQ; Yunnan, YN; and Guizhou, GZ; orange red), South (Guangxi, GX; Guangdong, GD and Hainan, HaN; dark green), and West (Xinjiang, XJ; Qinghai, QH; and Xizang, XZ; light purple). The red portion in each pie chart indicates the isolation rate of AIV in this region. The standard map was downloaded from Ministry of Natural Resources of the People’s Republic of China (http://bzdt.ch.mnr.gov.cn/), and the collection sites of LPMs in our study were marked on the map using ArcGIS. **b** AIV positive rates of the present study (2016–19) and the previous study in 2014–16^13^. The regions included North, East-Central, South-Central, Yangtze River Delta, South-West, and South. The numbers on the column represent the AIV isolation rate. **c** Subtype proportions of AIVs in the pure isolates with a single HxNy subtype. **d** The proportion of HA and NA from the impure isolates containing over two HA or NA subtypes. Source data are provided as a Source Data file.

A total of 16,091 samples were collected from May 2016 to February 2019 from 37 cities in 23 provinces, municipalities and minority autonomous regions in China (Fig. 1a), in which 2048 samples were identified to be AIV positive by next generation sequencing (NGS), with a positive rate of 12.73% (Supplementary Data 1–5).

To better analyze the geographical distribution of AIVs in LPMs in China, 23 provinces were divided into seven different regions on the basis of geographic proximity: North (Inner Mongolia, NM; Jilin, JL), East-Central (Shanxi, SX; Ningxia, NX; Shandong, SD; Shaanxi, SaX; Henan, HeN), South-Central (Anhui, AH; Hunan, HuN; Jiangxi, JX; Fujian, FJ), Yangtze River Delta (Jiangsu, JS; Zhejiang, ZJ), South-West (Sichuan, SC; Chongqing, CQ; Yunnan, YN; Guizhou, GZ), South (Guangxi, GX; Guangdong, GD; Hainan, HaN), and West (Xinjiang, XJ; Qinghai, QH; Xizang, XZ). The AIV positive rates in the seven regions were 5.01%, 9.50%, 20.48%, 20.83%, 8.11%, 8.80%, and 4.84%, respectively (Fig. 1a, b). Remarkably, aside from the East-Central region, the AIV positive rates in the other five regions (the North, South-Central, Yangtze River Delta, South-West, and South) substantially decreased during 2016–19 compared to those during 2014–16, especially in the South (from 32.40% to 8.80%) and South-West (from 31.78% to 8.11%) regions (Fig. 1b).

Further analysis revealed that the AIV isolation rate in ducks was the highest (17.88% [525 positive/2,936 samples]), followed by geese (14.52% [63/434]), chickens (12.47% [1,290/10,344]), environmental samples (9.29% [144/1,550]), and then pigeons (3.14% [26/827]) (Supplementary Data 1–5). The results demonstrated that the AIV isolation rates were higher in waterfowl (ducks and geese) than those of land poultry (chickens and pigeons).

### H9N2 AIV is dominant in LPMs in China

The 2048 AIV-positive isolates were then sequenced using NGS. The isolates with single HxNy subtype (pure isolates) were found in 70.41% (1442/2048) of the 2048 samples, and the isolates with over two HA or NA subtypes (impure isolates) were found in the remaining 29.59% (606/2,048) of the samples (Supplementary Data 1–5). Among the 1442 pure viruses with the AIV subtype clearly determined, H9N2 was the dominant subtype (*n* = 1049, 72.75%), with the proportions ranging from 57.69% (West) to 95.95% (East-Central) in the seven defined regions (Fig. 1c and Supplementary Data 1–5). However, the proportion of subtype composition and the prevalent subtype in the seven regions were slightly different. The isolation rates of H5 subtypes were higher in the North (33.33%) and in the West regions (42.31%: 30.77% for H5N8 and 11.54% for H5N6) compared to those in other regions (Fig. 1c). However, only 6 and 26 pure isolates were identified in the North and West regions, respectively (Supplementary Data 1–5).

H7N9 AIVs mainly circulated in the Yangtze River Delta with an isolation rate of 14.29% and in the South-Central region with 9.54%. H6N6 was prevalent primarily in three adjacent regions (South-Central, South, and South-West), with isolation rates between 6.20% and 9.32%. Overall, the top four subtypes circulating in LPMs in China included H9N2 (72.75%), H5N6 (7.84%), H7N9 (5.89%), and H6N6 (5.20%), respectively (Fig. 1c), and H9N2 has become dominant in LPMs in both Northern and Southern China.

The proportion of each specific HA and NA gene in the impure AIV isolates based on the NGS results were also analyzed. The top five HA and four NA subtypes for the impure isolates were H9 (41.15%), H5 (25.10%), H6 (14.49%), H3 (8.66%), H7 (7.39%), and N2 (45.14%), N6 (39.35%), N8 (6.31%), and N9 (4.91%), respectively (Fig. 1d and Supplementary Fig. 1a, b). The H9 and N2 were the dominant HA and NA subtypes, respectively. Meanwhile, the proportions of H9 and H5 subtypes in the 71 impure isolates were 42.25% and 7.04% in the North region, respectively, and 43.48% and 41.30% in the 92 impure samples in the West region (Supplementary Data 1–5). It should be noted that a few rare HA and NA subtypes such as H1, H3, H4, H10, H11, N1, N3, and N4 were observed in the impure samples, and a number of rare subtypes, such as H7N2/N3/N6/N7, H6N2/N8, and H9N6/N9, were also identified from the pure isolates (Fig. 1c and Supplementary Fig. 1c).

Therefore, there was a similar trend in the proportion of each HA and NA subtype between the pure and impure isolates, and the dominant HA and NA subtypes were the same in both groups. The top five HA subtypes in the pure isolates were H9 (74.41%), H5 (8.67%), H7 (8.25%), H6 (5.76%), and other (2.91%), whereas those in the impure isolates included H9 (41.15%), H5 (25.10%), H6 (14.49%), H7 (7.39%), and other (11.87%). The top five NA subtypes in the pure isolates were N2 (73.86%), N6 (13.73%), N9 (7.28%), N8 (1.87%), and other (3.26%), while those in the impure isolates were N2 (45.14%), N6 (39.35%), N8 (6.31%), N9 (4.91%), and other (4.29%) (Fig. 1d and Supplementary Data 1–5).

**Fig. 2:**
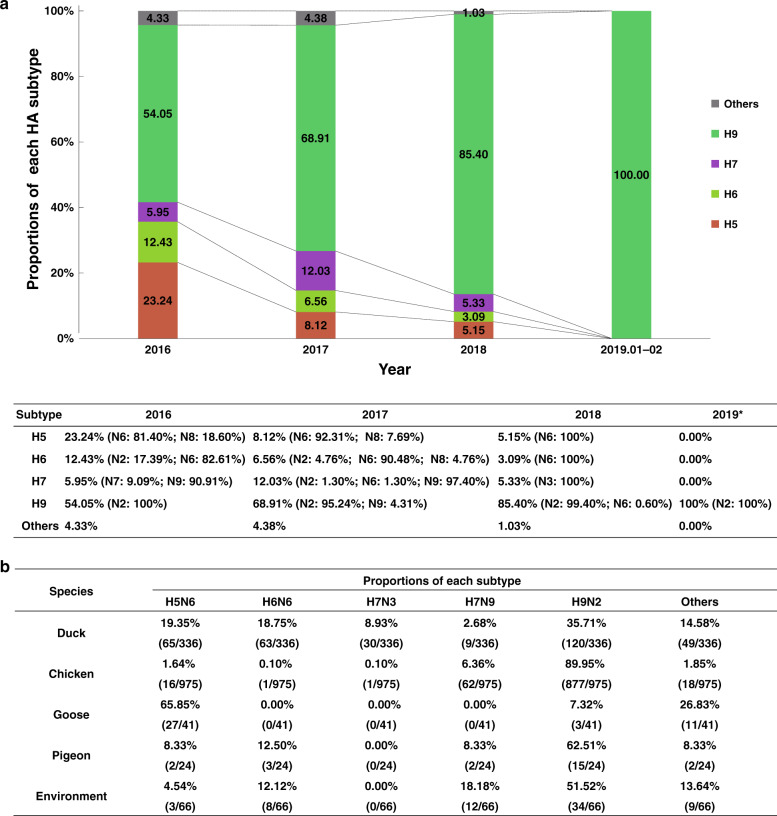
Virus subtype proportions and host species distributions of the pure isolates with a single HxNy subtype. **a** The proportion of various HA subtypes of pure isolates with a single HxNy subtype isolated between 2016 and 2019. The major prevalent subtypes include H5, H6, H7, and H9. H5 viruses include H5N6 and H5N8; H6 viruses include H6N2, H6N6, and H6N8; H7 viruses include H7N2, H7N3, H7N6, H7N7, and H7N9; H9 viruses include H9N2, H9N6, and H9N9. **b** Host species distributions of H5N6, H6N6, H7N3, H7N9, H9N2, and other subtypes. Source data are provided as a Source Data file.

To explore the distribution of virus subtypes in LPMs over the last few years, the subtype composition based on the NGS results between 2016 and 2019 was analyzed (Fig. 2a and Supplementary Fig. 1a–c). From 2016 to 2018, the proportion of H9N2 AIVs in the pure isolates steadily increased (54.05% in 2016, 65.63% in 2017, and 84.88% in 2018; Supplementary Fig. 1c). All 35 pure isolates from four provinces (Anhui, Henan, Shandong, and Shanxi) belonged to H9N2 during January and February of 2019 (Fig. 2a and Supplementary Data 1–5), and the H9 subtype was also found in 51.11% of the impure isolates. However, the proportion of pure H5Ny, H6Ny, and H7Ny AIVs decreased from 2016 to 2018 (Fig. 2a). Notably, the proportion of H7N9 isolates reached the peak in 2017 (11.72%), but almost disappeared in 2018 (Supplementary Fig. 1c), with just one H7N9 impure isolate identified containing H7 (151,233 reads), N9 (62,015 reads), and H9 (485 reads) gene sequences. Alternatively, H7N3 AIVs were identified in 2018 with a proportion of 5.33% (Fig. 2a and Supplementary Fig. 1c).

Our previous study has shown that different host species carried distinct major AIV subtypes^13^. As shown in Supplementary Fig. 2a, both H9N2 and H7N9 AIVs were mainly isolated from chickens (83.61% vs. 72.95%), and both H6N6 and H5N6 AIVs were mostly isolated from ducks (83.99% vs. 57.51%). Additionally, most of the rare subtypes were primarily isolated from ducks (65.84%). In contrast to our previous report that H9N2 and H5N6 were dominant in chickens and ducks, respectively, H9N2 was the prevalent subtype in both chickens (89.95%) and ducks (35.71%; Fig. 2b and Supplementary Fig. 2b). It should also be noted that for the three subtypes (H9N2, H7N9, and H5N6), more strains were isolated from the oropharyngeal swabs of chickens or ducks (50.05%, 17.65%, and 23.89%) than those from cloacal samples (16.97%, 4.71%, and 15.04%). Regarding the H6N6 subtype, strains isolated from cloacal samples (41.33%) were more than those from oropharyngeal swabs of ducks (25.33%; Supplementary Fig. 2a).

### Genetic evolution of H9N2 AIVs

**Fig. 3:**
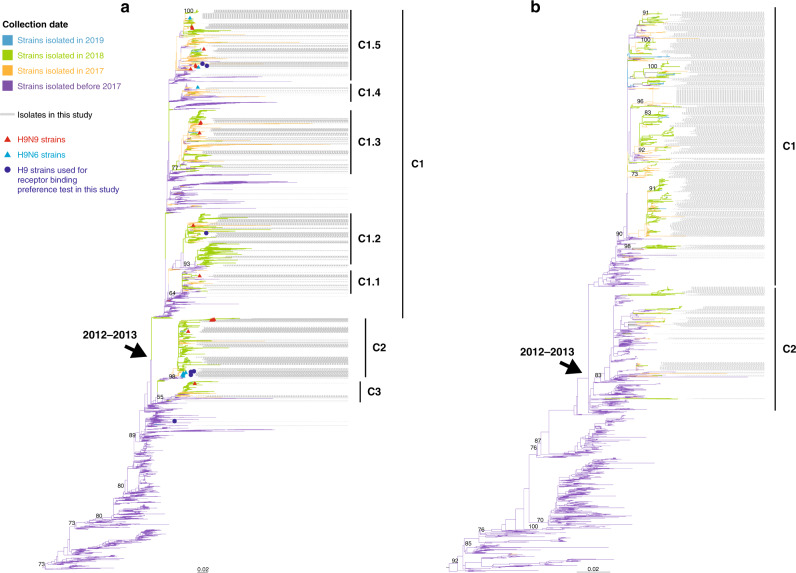
Phylogenetic analysis of the HA gene of H9Ny AIVs and the NA gene of H9N2 AIVs. **a** Phylogenetic tree of the HA gene of H9Ny AIVs. **b** Phylogenetic tree of the NA gene of H9N2 viruses. Viruses are marked with different colors according to the collection dates (before 2017: blue violet, in 2017: orange, in 2018: light green, and in 2019: light blue). Both trees are rooted using CK/BJ/1/1994(H9N2). The light blue and red triangles represent H9N6 and H9N9 viruses, respectively. All blue dots in the phylogenetic trees (**a**) represent H9N2 and H9N9 strains used for the receptor-binding test in this study. The labels with gray lines indicate all of the H9 isolates sequenced in this study.

Since H9N2 AIVs have now become dominant in China, we performed a phylogenetic analysis of 7521 HA genes of H9N2 AIVs from China, including 1477 sequences described in the present study (Fig. 3a). The HA phylogenetic tree revealed that Chinese H9N2 AIVs diverged approximately during 2012–13, resulting in three Clades (C1–C3) with between-group distance of ≥1% (Supplementary Data 6). C1 continued to diverge into several highly similar small sub-clades C1.1–C1.5 (with between-group distance of ≥0.3%, Supplementary Data 6), and most have been co-circulating during our surveillance period. In contrast, C2 and C3 viruses circulated at very low levels, with few viruses isolated from 2012 to 2016. However, the prevalence of C2 and C3 remarkably increased during 2017 and 2018. In detail, 2498 H9 strains isolated since 2017 belonged to C1, 783 viruses belonged to C2, and 211 belonged to C3. Regarding our H9 isolates since 2017 (*n* = 1350), 937 strains fell within C1, 393 in C2, and 20 in C3.

Although the majority of the H9 isolates belonged to the H9N2 subtype, several H9N9 and H9N6 viruses were also found to be co-circulating, scattering amongst the tree with H9N2 AIVs, without forming separate clusters (Fig. 3a). Similarly, in the NA phylogenetic tree of the H9N2 AIVs, there were two major Clades (C1 and C2, with between-group distance of ≥1.5%, Supplementary Data 6), and they also diverged during 2012–13 (Fig. 3b). The majority of our isolates since 2017 (*n* = 1151) belonged to C1 and 161 isolates belonged to C2. Therefore, multiple clusters of H9N2 AIVs have been co-circulating in China after 2012–13.

Several amino acids (Q226L, I155T, and H183N) affecting the receptor-binding preference of H9Ny AIVs were analyzed. The majority of the H9 strains possessed 226L (99.93% [1,438/1,439]), 155T (99.58% [1,433/1,439]), and 183N (99.93%, [1,438/1,439]; Supplementary Data 7 and 8), suggesting that they may have acquired human receptor (α2-6-SA) binding capacity. In total, 99.43% (1395/1403) of the sequenced H9N2 strains had NA stalk deletions (positions 62–64), and no NA inhibitor (NAI)-resistant mutations were found in the NA proteins of H9Ny (Supplementary Data 7).

### Continued evolution and emergence of H5, H7, and H6 variants

**Fig. 4:**
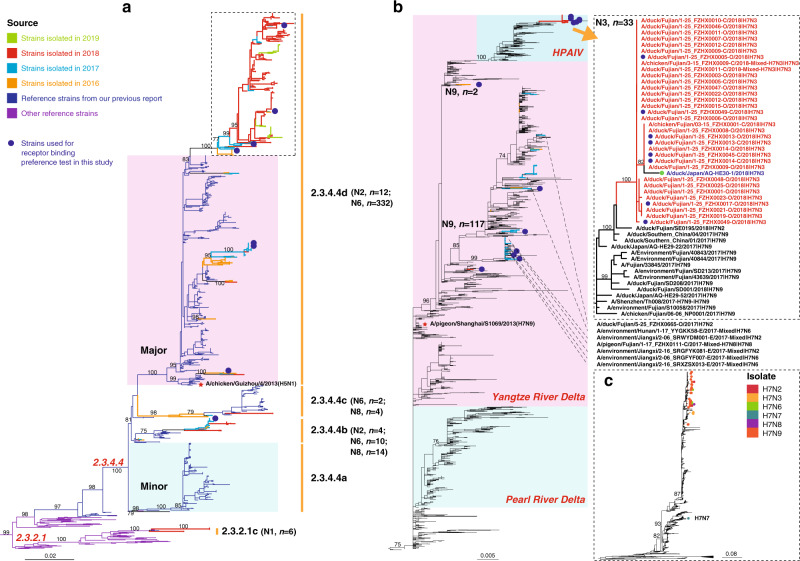
Phylogenetic analysis of the HA gene sequences of H5 and H7 AIVs. **a** Phylogenetic tree of the HA gene of H5 AIVs. The orange, light blue, red, and light green lines in the tree represent viruses described in this study isolated from 2016, 2017, 2018, and 2019, respectively. The dark blue lines represent the reference strains previously reported by Bi et al.^13^. The subtrees marked with a pink and light blue background represent the major lineage (Clade 2.3.4.4d) and the minor lineage (Clade 2.3.4.4a), respectively. The purple lines represent other reference strains from the Influenza Virus Resource at NCBI and the GISAID databases. **b** Phylogenetic tree of the HA gene of H7 AIVs. The subtrees marked with a pink and light blue background represent H7 strains belonging to the Yangtze River Delta lineage and Pearl River Delta lineage, respectively. The subtree of the H7N9 HPAIVs previously analyzed by Quan et al.^23^ is marked with the blue background on the upper right. The orange, light blue, and red lines of the tree represent strains isolated from 2016, 2017, and 2018, respectively. The subtree displayed in the dashed frame on the upper right included the HA genes of 33 H7N3 isolates in this study. The dotted lines represent H7N2 (*n* = 3), H7N6 (*n* = 3), and H7N8 (*n* = 1) viruses identified in this study. **c** The topology of the HA tree of H7 AIVs was shown at the bottom-right, with dots represent 160 H7 AIV strains identified in our surveillance during 2016–19. All blue dots in the phylogenetic trees (**a**, **b**) represent the H5 and H7 strains used for receptor-binding test in this study. In addition, the red pentagrams represent the H5/H7 bivalent vaccine strains, A/chicken/Guizhou/4/2013(H5N1) and A/pigeon/Shanghai/S1069/2013(H7N9), respectively.

The H5 subtype was detected in 384 of the isolates sequenced from 2016 to 2019. H5N6 subtype AIVs (*n* = 344) accounted for the majority (89.58%), followed by H5N8 (*n* = 18, 4.69%) and H5N2 (*n* = 16, 4.17%). All of the H5N6, H5N8, and H5N2 AIVs fell within Clade 2.3.4.4 (Fig. 4a), which could be classified into four sub-clades, with between-group distance of ≥3% (Supplementary Data 6). Clades 2.3.4.4a and 2.3.4.4d corresponded to the minor and major lineages designated in our previous report^13^. Clades 2.3.4.4b and 2.3.4.4c were already found to exist in our previous research, but were not designated then. In total, 344 strains, including 332 H5N6 and 12 H5N2 AIVs, fell within Clade 2.3.4.4d (the previously designated major lineage), whereas 28 strains (H5N2, *n* = 4; H5N6, *n* = 10; H5N8, *n* = 14) clustered in 2.3.4.4b and 6 strains (H5N6, *n* = 2; H5N8, *n* = 4) clustered in 2.3.4.4c. Notably, none of our strains belonged to Clade 2.3.4.4a (the previously designated minor lineage). Although 344 strains were classified into 2.3.4.4d, most (*n* = 324) formed a separate sub-clade within 2.3.4.4d with a distance of 1.2%. In addition, >80% strains in this unique sub-clade possessed distinct amino acid substitutions in the HA antigenic regions according to the H3 structure^24–26^. It should be noted that only six strains from 2018 belonged to the H5N1 subtype, and all of them fell within Clade 2.3.2.1c (Fig. 4a).

Our surveillance identified a total of 160 H7 AIV strains during 2016–19. To our surprise, they belonged to at least six different subtypes: H7N9 (*n* = 119), H7N3 (*n* = 33), H7N2 (*n* = 3), H7N6 (*n* = 3), H7N8 (*n* = 1), and H7N7 (*n* = 1; Fig. 4b, c). Phylogenetic analysis of the HA gene showed that apart from one H7N7 strain Dk/JX/1-07 NCDZT35N-C/2016, all of the remaining H7 strains (*n* = 159) clustered together with the human-infecting H7N9 AIVs (Fig. 4c) within the Yangtze River Delta lineage. Apart from H7N3, other H7 subtypes, e.g. H7N2, H7N6, and H7N8 AIVs scattered in the Yangtze River Delta lineage with LPAIV H7N9 contemporarily circulating in LPMs in different regions of China (Fig. 4b).

Of note, 31 of 33 H7N3 strains isolated from ducks and two strains from chickens from Fujian in 2018 formed an independent cluster within the H7N9 HPAIV lineage (Fig. 4b). The NA gene sequences of the H7N3 AIVs showed that they were closely related to AIVs of the N3 subtype circulating in ducks in southern China during 2017–18 (Supplementary Data 9). We have performed a complete phylogenetic analysis of the eight gene segments of 615 H7N3 AIVs (584 strains from public databases). Our analyses revealed that the H7N3 AIVs had diverged into the North American lineage and the Eurasian lineage. These strains could be further classified into 26 genotypes (Supplementary Fig. 3 and Supplementary Data 10), with 14 belonging to the North American lineage and 12 belonging to the Eurasian lineage (Supplementary Fig. 3 and Supplementary Data 9 and 10). Our 31 H7N3 AIVs with whole genome (two isolates only had HA sequences) described here belonged to G11 (*n* = 30) and G12 (*n* = 1), respectively. They were different from a reassortant H7N3 strain identified from Japan, A/duck/Japan/AQ-HE30-1/2018(H7N3)^27^ (G10), in the PB2 gene. Dk/FJ/1.25 FZHX0009-C/2018(H7N3) (G12) differed from the remaining 30 H7N3 strains (G11) in the MP and NS genes. Therefore, all of these H7N3 strains were not closely related to H7 AIVs from other countries, such as Mexico and the USA, and were novel reassortants.

All of the H5 viruses (*n* = 384) described in the present study possessed multiple basic amino acid residues at the cleavage site, whereas the H7N9 and H6Ny LPAIVs had less basic amino acids (PKGRGL or PQIETRGL). All of the H7N3 viruses (*n* = 33) also possessed multi-basic cleavage sites (PKRRRTARGL). Regarding the receptor-binding associated sites, 100% (401/401) of the H5 viruses had 226Q (H3 numbering). In all, 73.89% (116/157) of the H7 AIVs had 226L, and 99.36% (156/157) had 186V. All of the 33 H7N3 isolates possessed 186V and 226Q. Almost all 169 H6 strains possessed 190E, 226Q, and 228G (Supplementary Data 7).

### Receptor-binding properties of the major AIVs

In order to identify and provide early-warning of the potential public risks of the AIVs, a total of 43 representative strains including H9N9 (*n* = 7), H9N2 (*n* = 3), H5N6 (*n* = 8), H7N9 (*n* = 7), H7N3 (*n* = 9), H6N6 (*n* = 5), and H6N2 (*n* = 4) were selected for receptor-binding test using trisaccharide receptors.

**Fig. 5:**
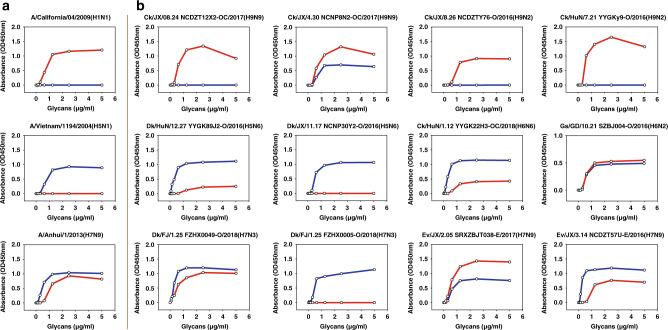
Receptor-binding properties of representative AIV isolates. **a** A/Anhui/1/2013(H7N9) was used as a reference for comparison with the tested H7 strains. Two human strains, A/California/04/2009(H1N1) and A/Vietnam/1194/2004(H5N1), were used as controls. **b** Receptor-binding properties of the representative AIV strains to human (α2-6-SA) and avian (α2-3-SA) receptors were tested using the solid-phase direct binding assay with trisaccharide receptors. Red and blue lines represent human- and avian-type receptors, respectively. Two replications presented similar results and the mean values were shown. Source data are provided as a Source Data file.

All tested H9 isolates possessed residue 226L (Supplementary Data 7). As expected, six H9N9 and two H9N2 testing strains (Ck/JX/08.24 NCDZT12X2-OC/2017(H9N9), Ck/JX/4.30 NCDZT44N2-OC/2017(H9N9), Ck/JX/4.30 NCDZT59N2-OC/2017(H9N9), Ck/JX/08.24 NCDZT49X2-OC/2017(H9N9), Ck/JX/6.26 NCDZT51R2-OC/2017(H9N9), Ck/JX/4.30 NCDZT36N2-OC/2017(H9N9), Ck/JX/8.26 NCDZTY76-O/2016(H9N2), and Ck/HuN/7.21 YYGKy9-O/2016(H9N2)) exclusively bound to human-type receptors (α2-6-SA). Only one H9N9 strain (Ck/JX/4.30 NCNP8N2-OC/2017(H9N9)) and one H9N2 (Ck/GD/4.18 SZBJ011-O/2018(H9N2)) presented a dual receptor-binding ability, with preference for human-type receptors (Fig. 5, Supplementary Fig. 4a, and Supplementary Data 8).

All tested H5N6 strains possessed 226Q and a loss of glycosylation site at the positions 158–160 (Supplementary Data 7), which mainly bound to avian-type receptors. As expected, the four tested strains (Dk/HuN/12.27 YYGK89J2-O/2016(H5N6), Gs/XJ/11.29 WLMQXL001-O/2017(H5N6), Gs/FJ/10.26 FZHX0002-C/2017(H5N6) (mixed Q (57.82%) and R (41.76%) at position 227), and Ck/SD/2.28 TAWM016-C/2017(H5N6)) displayed weak affinities to human-type receptors (Fig. 5, Supplementary Fig. 4a, and Supplementary Data 8).

All seven H7N9 strains were found to possess the ability to bind both avian and human-type receptors. It was notable that four strains (Ev/JX/2.05 SRXZBJT038-E/2017(H7N9) (186V and 226L; mixed R (49.59%) and K (49.27%) at position 173), Ev/JL/04.11 CCHSL037-E/2018(H7N9) (186V and 226I), Ev/JX/2.16 SRGFYK089-E/2017(H7N9) (186V and 226L), and Ev/JX/1.11 NCDZT98F2-E/2017(H7N9) (186V, 226L, 122T, and 236I)) preferred binding to human-type receptors compared to the precursor A/Anhui/2013(H7N9) and other tested strains with 186V and 226L (Fig. 5, Supplementary Fig. 4a, and Supplementary Data 8), suggesting that the transmissibility from avian to humans may have increased for these H7N9 isolates. For the H7N3 reassortants with HA gene from H7N9 HPAIV, six of the nine strains (Dk/FJ/1.25 FZHX0049-O/2018(H7N3), Dk/FJ/1.25 FZHX0017-O/2018(H7N3), Dk/FJ/1.25 FZHX0011-O/2018(H7N3), Dk/FJ/1.25 FZHX0045-C/2018(H7N3), Dk/FJ/1.25 FZHX0014-C/2018(H7N3), and Dk/FJ/1.25 FZHX0046-O/2018(H7N3)) bound to both avian and human-type receptors and the affinities to avian-type receptors were slightly stronger than those to human-type receptors, whereas three strains (Dk/FJ/1.25 FZHX0005-O/2018(H7N3), Dk/FJ/1.25 FZHX0013-O/2018(H7N3), and Dk/FJ/1.25 FZHX0013-C/2018(H7N3)) only bound to avian-type receptors (Fig. 5, Supplementary Fig. 4, and Supplementary Data 8).

For the H6 subtype, the tested strains also displayed diverse receptor-binding abilities. Three H6N6 strains with 190E and 228G (Dk/HuN/2.06 YYGK86J3-OC/2018(H6N6), Dk/JX/5.28 NCNP34N3-OC/2018(H6N6), and Dk/HuN/5.29 YYGK100P3-OC/2018(H6N6)) only bound to avian-type receptors. However, another two H6N6 strains also with 190E and 228G (Ck/HuN/1.12 YYGK22H3-OC/2018(H6N6) and Dk/HuN/11.30 YYGK54E3-OC/2018(H6N6)) possessed both avian- and human-type receptor-binding abilities and preferred avian-type receptors (Fig. 5, Supplementary Fig. 4, and Supplementary Data 8). In contrast to H6N6, all four H6N2 representative strains possessed double receptor-binding abilities. Gs/GD/10.21 SZBJ001-O/2016(H6N2) (190V and 228G) and Gs/GD/10.21 SZBJ001-C/2016(H6N2) (190V and 228G) possessed higher affinities to avian-type receptors, while Gs/GD/10.21 SZBJ004-O/2016(H6N2) (190A, 222I, and 228G) and Gs/GD/10.21 SZBJ003-O/2016(H6N2) (190V and 228S) displayed a preference for human-type receptors (Fig. 5, Supplementary Fig. 4, and Supplementary Data 8).

The predominance of human-type receptor-binding preference of the tested H7N9, H9N2, and H9N9 strains was further confirmed using pentasaccharide receptors (Supplementary Fig. 4b). The receptor-binding affinities to both trisaccharide and pentasaccharide receptors were also similar for the tested strains of other subtypes, although Dk/FJ/1.25 FZHX0005-O/2018(H7N3) and Dk/HuN/2.06 YYGK86J3-OC/2018(H6N6) displayed slight binding avidities to human-type pentasaccharide receptors, compared with single affinities to avian-type trisaccharide receptors (Fig. 5, Supplementary Fig. 4, and Supplementary Data 8). These data indicated that many H5, H6, H7, and H9 AIVs have acquired a capability for binding to human-type receptors.

## Discussion

Compared to our previous study^13^, the AIV positive rates substantially decreased from 2016 to 2019. Several factors may have accounted for this decline. Due to LPMs as a transmission source and even potential incubator for human infections with AIV^28,29^, more and more provinces have started to close LPMs^30–32^ or take special measures at the human-animal interface to lower the risks of human infection. For example, the “1110” strategy for markets (cleaning every day, disinfecting every week, shutting down once per month, and butchering all unsold live birds before closing every day) was first proposed and implemented in Guangdong Province, China in 2014 (http://www.chinanews.com/fz/2014/12-06/6851778.shtml). Similar strategies have since been implemented in other Chinese provinces. In 2018, the Ministry of Agriculture of China required all Chinese provinces to implement the “1110” strategy (http://www.moa.gov.cn/nybgb/2018/201803/201805/t20180528_6143196.htm). In addition, the H5/H7 bivalent vaccine may have also contributed to the decreased AIV positive rates^33^. However, after using the H9 and H5 vaccines for ~21^34^ and 15^35^ years, respectively, these viruses are still circulating and evolving in China. Although the factors contributing to the decreased AIV positive rates in China need to be further investigated, the “1110” strategy may be effective, and lower AIV positive rates in LPMs would be expected as a result.

Since 2016, the dominant AIV subtypes have substantially changed. During 2014–16, H9N2 and H5N6 were the dominant subtypes in Northern and Southern China, respectively^13^. However, the proportion of H9N2 AIVs gradually increased and has now become the most prevalent subtype in both Northern and Southern China. Coupled with the nation-wide and disordered transportation of poultry carrying H9N2, the emergence of H9N9 and H9N6 reassortants, and the dynamic reassortments among H9 and different AIV subtypes^14,16^, the circulation of H9 LPAIVs has become highly complicated in China. Remarkably, despite the widespread circulation of H7N9 AIVs during 2016–17^23^, it almost disappeared in 2018. The shift of the AIV subtypes in the poultry was not likely associated with intraspecies transmission between chicken and ducks, but may be caused by changes in the management of LPMs, the vaccination strategy, and different sensitivities of various viruses to the disinfectants used in the “1110” strategy. However, these results highlight the distinct change of the dominant AIV subtypes in China and will have a profound influence on prevention strategies against AIVs, including vaccine development and usage.

Moreover, the emergence of a number of variants was notable, especially the H7N3 variant with an HA gene of the H7N9 HPAIV origin and the H5N6 variant. The antigenicity of these mutants, the effectiveness of the current H5/H7 bivalent vaccine against these variants, as well as the reason for H7N9 being replaced by H7N3, warrant further investigation. In addition, AIV isolation rates in the Yangtze River Delta and the South-Central regions only slightly decreased and were still higher than 20.00%. The co-infection or “impure” AIVs may also lead to antigenic or subtype shift. Including the present study, the existence of impure isolates with different subtypes has also been reported in many studies^36,37^. All the results highlight the necessity of constant surveillance of AIVs in LPMs.

It is known that five out of the 12 AIV subtypes that have been detected in cases of human infections are H7 subtypes, including H7N2, H7N3, H7N4, H7N7, and H7N9^3,10,38–40^. In this study, we revealed that five H7 (H7N2, H7N3, H7N6, H7N7, and H7N9) subtypes were co-detected in LPMs, in which most were isolated from ducks (except for H7N9), suggesting that ducks may also act as a “mixing vessel” for the H7 AIV reassortants. In fact, most of the other rare subtypes with diverse genetic constellations were also found in ducks. This may be partly due to the more contacts between domestic duck and wild waterfowl, which was considered as the natural reservoir of AIVs. H9N2 has also become the major subtype in ducks. Therefore, the probability of emergence of novel AIVs by reassortment among the diverse genetic constellations may likely be higher in ducks, not only because of the high diversity of AIV genetic constellation in ducks but also the excellent genetic compatibilities among H9N2 and other influenza subtypes including H7N9, pandemic H1N1, H5N1, H5N6, and so on^13,16,41–43^. Therefore, several AIV subtypes potentially infecting humans were circulating in LPMs and intensive surveillance of AIVs particularly among ducks should be performed continuously.

Receptor binding was considered as the first step of influenza infection to host cells^44–47^. Almost all H9Ny isolates possessed 226L on HA, and all the tested H9N2 and H9N9 strains mainly bound to human-type receptors. 96.58% (113 out of 117) of the H7N9 isolates possessed both 186V and 226L, which were considered as the critical sites for human-type receptor binding of H7N9 AIVs^46,48,49^, and all the tested H7N9 strains presented affinities to both avian- and human-type receptors. Notably, several representative H7N9 strains during 2017–18 preferred human-type receptors, and the binding avidities were much stronger than a previous H7N9 strain (A/Shenzhen/Th001/2016), which also preferred to bind human-type receptors^50^. Six of the nine tested H7N3 HPAIVs displayed dual receptor-binding abilities though preference to avian-type receptors. This phenomenon was also seen in the H7N9 HPAIVs^48,50,51^, indicating potentially similar infectivity of H7N3 and H7N9 HPAIVs to the hosts.

All the sequenced H5N6 strains had 226Q, while some tested strains presented slight preference for human-type receptor, which could be partly explained by the loss of a glycosylation site at the positions 158–160^52–54^. E190V and G228S mutations on HA contributed to the human-type receptor-binding abilities for H6 viruses^55,56^. Although almost H6 strains possessed 190E and 228G, several H6N2 strains were found to have E190V and/or G228S mutations, which could explain the dual receptor-binding ability. However, H6N6 strains with 190E and 228G were also found to bind to both receptors. Taken together, our receptor-binding tests highlight that only few AIV strains showed pure binding abilities to avian-type receptors, whereas the majority presented human-type receptor-binding capacity, particularly the dominant H9 AIVs. Therefore, despite lower positive rate in LPMs, AIVs showed increased abilities and risk to infect humans, which deserves closer attention.

In summary, our latest nation-wide AIV surveillance data revealed a decrease of AIV positive rate and H9N2 has become the prevalent subtype throughout China. Most AIVs have obtained human-type receptor-binding abilities, including H5, H6, H7, and H9 subtypes, in which the H7N9 and H6N2 variants and almost all H9Ny strains preferred binding to human-type receptors. Furthermore, mutations associated with antigenic variation have been found in the H7N9, H7N3, and H5N6 variants. In fact, sporadic human cases caused by H7N9, H5N6, and H9N2 continue to be reported^5,57^, and the seroprevalence rate of H9N2 AIVs in the poultry workers posed an increasing trend after 2009 in China^7,58^. Therefore, constant monitoring on AIVs should be more closely conducted for agricultural and public health.

## Methods

### Eggs

Embryonated chicken eggs obtained from Beijing Vital River Laboratory Animal Technology Company were incubated at 37 °C and 80% humidity for 10 days before being used for virus isolation.

### Sample collection and virus isolation

Oropharyngeal and cloacal swabs from apparently healthy poultry, as well as environmental samples, were collected in LPMs in 37 cities and counties across 23 provinces or municipalities or minority municipalities in China. Poultry included chickens, ducks, geese, and pigeons. Environmental samples included swabs from cages, poultry drinking water, defeathering machines, chopping boards, and feces in the LPMs. Sampling was collected from May 2016 to February 2019 (samples collected once a month, unless the LPM was closed, and there were no samples collected in the corresponding month), a period of 26 months spanning three flu seasons. Compared to our previous study, the same or nearby LPMs in Inner Mongolia, Jilin, Henan, Shandong, Jiangsu, Zhejiang, Hunan, Jiangxi, Anhui, Fujian, Guangdong, Guangxi, Sichuan, and Yunnan were chosen for sampling^13^. Furthermore, sampling was also performed in several additional provincial level administrative regions, including Xinjiang, Xizang, Qinghai, Guizhou, Hainan, Shaanxi, Shanxi, Ningxia, and Chongqing. The swabs were placed into viral transport media and transported to the laboratory within 24 h in a handheld portable 4 °C refrigerator, and frozen at −80 °C immediately for future use. Avian influenza viruses were isolated in 10-day-old specific pathogen-free (SPF) chicken embryos according to the WHO manual^59^. After culture, all the hemagglutinin-positive and -negative allantoic fluids were further tested by RT-PCR using universal primers^13^ targeting the PB1 and/or M gene as listed in Supplementary Data 11.

### Whole-genome sequencing of AIV isolates

Viral RNA was extracted directly from AIV-positive allantoic fluid with MagaBio plus Virus RNA Purification Kit (BIOER, China). The whole-genome of AIV isolates were sequenced using Next generation sequencing (NGS)^13^. Briefly, RT-PCR and DNA synthesis were performed using the PrimeScript One Step RT-PCR kit (Takara). Next, the sequencing libraries were prepared. The libraries were sequenced on the BGI500 and Illumina HiSeq 4000. Sequencers by 200 bp or 250 bp paired-end sequencing, and sequencing depth for AIV isolates was about 0.2G per sample. The accuracy of the NGS method was confirmed by the published qRT-PCR method^60^ and qRT-PCR kits (Mabsky Biotech Co., Ltd.) with reference samples.

### Sequencing data assembly

Raw NGS reads were processed by filtering out low-quality reads (eight bases with quality <66), adapter-contaminated reads (with >15 bp matched to the adapter sequence), poly-Ns (with 8Ns), duplication and host contaminated reads (SOAP2 version 2.21; less than five mismatches)^13,61^. The filtered reads were mapped to the INFLUENZA database (downloaded on 1 June 2018)^62^ to choose best-matching reference sequences. Burrows-Wheeler Aligner (BWA version 0.7.12)^63^ and SAMtools (version 1.4)^64^ were then used to perform reference-based assembly.

Based on the NGS data, each cultured sample with ≥2 HA or NA subtypes was defined as “impure isolate”, while those with single HA and NA subtype were defined as “pure isolate”. The AIV positive samples are the cultured samples including both pure and impure isolates. The AIV positive rate was then calculated by “the numbers of AIV positive (cultured) samples” divided by “the total numbers of cultured samples”. The percentage of the impure or pure isolate was calculated as “the numbers of impure or pure isolates” divided by “the total number of AIV positive samples”.

### Phylogenetic analyses

Complete genomes of the AIVs isolated in China were downloaded from the Influenza Virus Resource at the National Center for Biotechnology Information (https://www.ncbi.nlm.nih.gov/genomes/FLU/Database/nph-select.cgi)^62^ and the GISAID (https://www.gisaid.org/)^65^ database on 2019. Repetitive sequences in the two databases were removed by matching strain names using Bioedit (version 7.1.3.0)^66^. Only full-length genomes were kept and sequence with obvious errors (e.g. frameshifts or total number of ambiguous bases >100) were excluded manually. The remaining sequences were combined with those generated in the present study, and the sequences of H9Ny, H5Ny, H7Ny, and H6Ny isolates were phylogenetically analyzed.

Multiple sequence alignment was performed using Muscle (version 3.8.31)^67^ and then adjusted manually in Bioedit (version 7.1.3.0)^66^. Phylogenetic analysis of the aligned HA and NA datasets were performed using RAxML (version 8.1.6)^68^, with GTRGAMMA applied as the nucleotide substitution model with 100 bootstrap replicates. Trees were visualized using FigTree (version 1.4.3).

The H5 clades in the phylogenetic trees were defined according to the nomenclature system proposed by FAO/WHO/OIE (https://www.who.int/influenza/gisrs_laboratory/h5_nomenclature_clade2344/en/) and previous publications^13,69^. The classification of Yangtze River Delta and Pearl River Delta lineages in HA genes of the human-infecting H7N9 AIVs are defined based on previous publications^23,70,71^. The HA and NA clades of H9N2 were defined based on pairwise distance between taxa calculated with default parameters in MEGA (version 5.2)^72^. A HA or NA clade was defined when the between-group distance was ≥1%.

### Receptor-binding assay

The pure isolates in different HA clades, within three passages and presenting ≥64 HA titers, were selected as the representative strains for receptor-binding testing using the solid-phase direct binding assay^73^. Briefly, 96-well microtiter plates were coated with biotinylated glycans α2-3-SA receptors (trisaccharide: Neu5Acα2-3Galβ1-4GlcNAcβ-SpNH-LC-LC-Biotin and pentasaccharide: NeuAcα2-3Galβ1-4GlcNAcβ1-3Galβ1-4GlcNAcβ1-SpNH-LC-LC-Biotin) and α2-6-SA receptors (trisaccharide: Neu5Acα2-6Galβ1-4GlcNAcβ-SpNH-LC-LC-Biotin and pentasaccharide: NeuAcα2-6Galβ1-4GlcNAcβ1-3Galβ1-4GlcNAcβ1-SpNH-LC-LC-Biotin). Virus dilutions containing 64 HA units with the NAIs (10 μM each of Oseltamivir and Zanamivir) were incubated. Virus-receptor-binding was detected with rabbit antisera against the influenza viruses (H1, H5, H6, H7, or H9, CASCIRE)^74^ and HRP-linked goat-anti-rabbit antibody (Bioeasytech). HRP-linked goat-anti-rabbit antibody was diluted 2000 times in phosphate buffer saline (PBS) with 1% bovine serum albumin (BSA). The results were measured by tetramethylbenzidine (TMB) at 450 nm. A/Anhui/1/2013(H7N9) was used as a reference for comparison with the tested H7 strains. Two human strains, A/California/04/2009(H1N1) and A/Vietnam/1194/2004(H5N1) were used as control.

### Biosafety statement and facility

Routine surveillance samples were processed in the biosafety level 2 (BSL-2) labs of CASCIRE. Coveralls, gloves, and N95 masks were used during the working in BSL-2 labs, and all wastes were autoclaved. The experiments with live H7N9, H7N3, and H5N6 viruses were conducted in biosafety level 3 (BSL-3) labs of CASCIRE or CASCIRE Network Surveillance Unit (NSU). This study was approved by the Ethics Committee of Institute of Microbiology, Chinese Academy of Sciences (SQIMCAS2016016).

### Measures against cross-contamination

First, during the sample collection process, all the tubes with samples were placed into different cells in sample box. Second, our longitude study included >16,000 samples, however, samples were not detected at the same time. In fact, samples from the same site were detected as soon as possible after collection by month, and usually no more than 200 samples were identified in each experiment. Third, before the inoculation or identification of the original and cultured samples, the surfaces of tubes were disinfected with disinfectant (Benzalkonium bromide or 75% alcohol). Fourth, RNAs were extracted by an automatic nucleic acid purification machine (Nucleic Acid Purification System NPA-32) rather than manually. Fifth, all the experiments associated with original and cultured samples, as well as RNAs (sample handling, virus isolation, PCR system preparation, and NGS library preparation), were performed in biosafety cabinets with tweezers and tips with filters. Tubes with samples were centrifuged at 5000 × *g* for ~10 s, and then were opened using tweezers, which will be disinfected by flameless infrared heater after each usage. In addition, disposable coveralls, N95 marks (Zhuozhou Fumeishendun Biotechnology Co., Ltd., China), and double-deck gloves (the inner shorter gloves cover the cuff by adhesive tape, and the outer longer gloves also cover the cuff but without adhesive tape for easy changing if they were contaminated) were strictly dressed in each experiment.

## Supplementary information

Supplementary Infomation

Description of Additional Supplementary Files

Supplementary Data 1

Supplementary Data 2

Supplementary Data 3

Supplementary Data 4

Supplementary Data 5

Supplementary Data 6

Supplementary Data 7

Supplementary Data 8

Supplementary Data 9

Supplementary Data 10

Supplementary Data 11

Reporting Summary

## Data Availability

Data supporting the findings of this study are available within the article and its Supplementary Information files. The H9Ny, H5Ny, H7Ny, and H6Ny sequences reported in this paper have been deposited into Global Initiative on Sharing All Influenza Data databases (GISAID; https://www.gisaid.org), and the accession numbers are listed in the Supplementary Data 7. These sequences have also been deposited into GenBank (accession numbers MW094306 - MW110364) and the China National Microbiological Data Center (accession number NMDC10017696 and genome accession numbers NMDCN0000230 - NMDCN0000HOQ). Source data are provided with this paper.

## Source data

Source Data
