# Supplementary material for: Dominant subtype switch in avian influenza viruses during 2016–2019 in China
Source: Nat Commun. 2020 Nov 20;11:5909. doi: 10.1038/s41467-020-19671-3 (PMC7679419; doi:10.1038/s41467-020-19671-3)
Supplement: Supplementary file 1 — Supplementary Infomation [file 41467_2020_19671_MOESM1_ESM.pdf]

Supplementary Information

Dominant subtype switch in avian influenza viruses during 2016-2019 in China

Yuhai Bi et al.

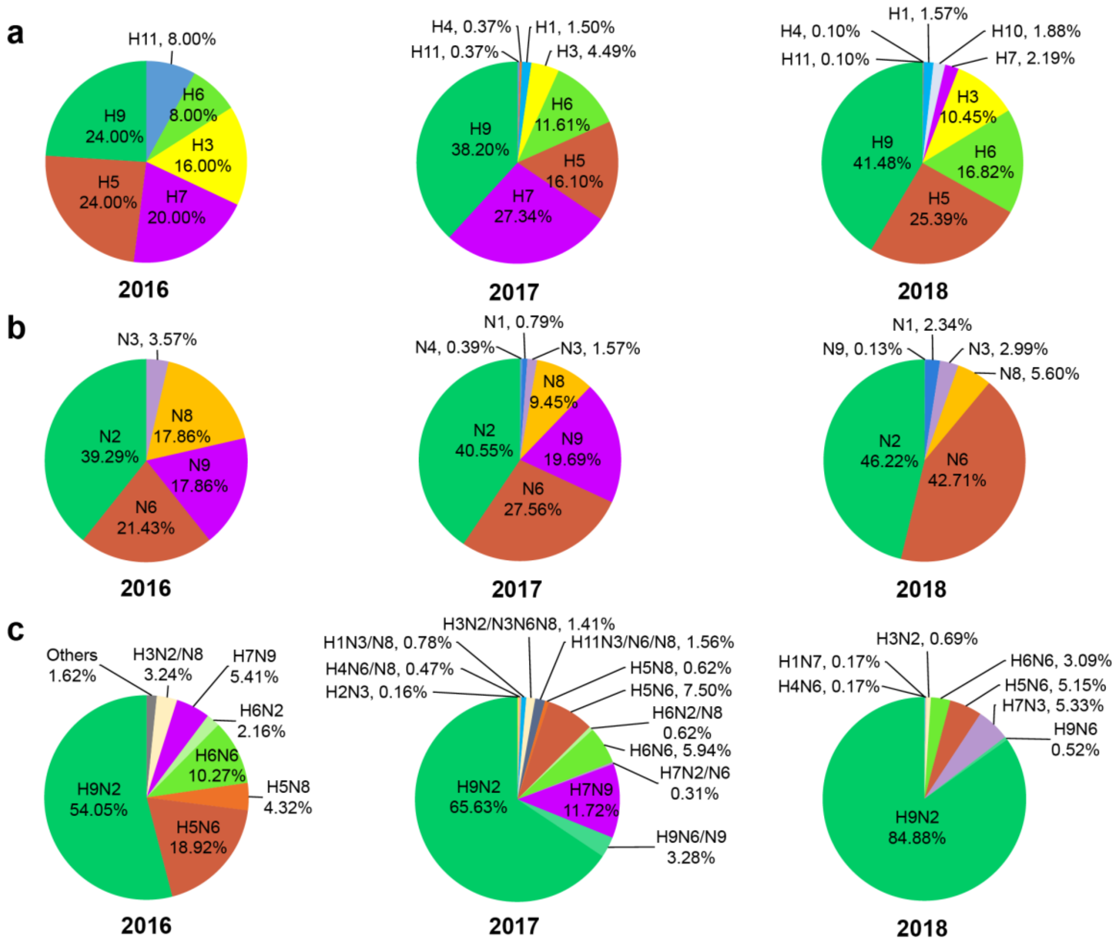

Supplementary Figure 1. Distribution of virus subtypes in LPMs between 2016 and 2018. (**a**) The HA subtype proportions of impure isolates with over two HA or NA subtypes. (**b**) The NA proportions of impure isolates with over two HA or NA subtypes. (**c**) Subtype proportions of pure isolates with a single HxNy. Source data are provided as a Source Data file.

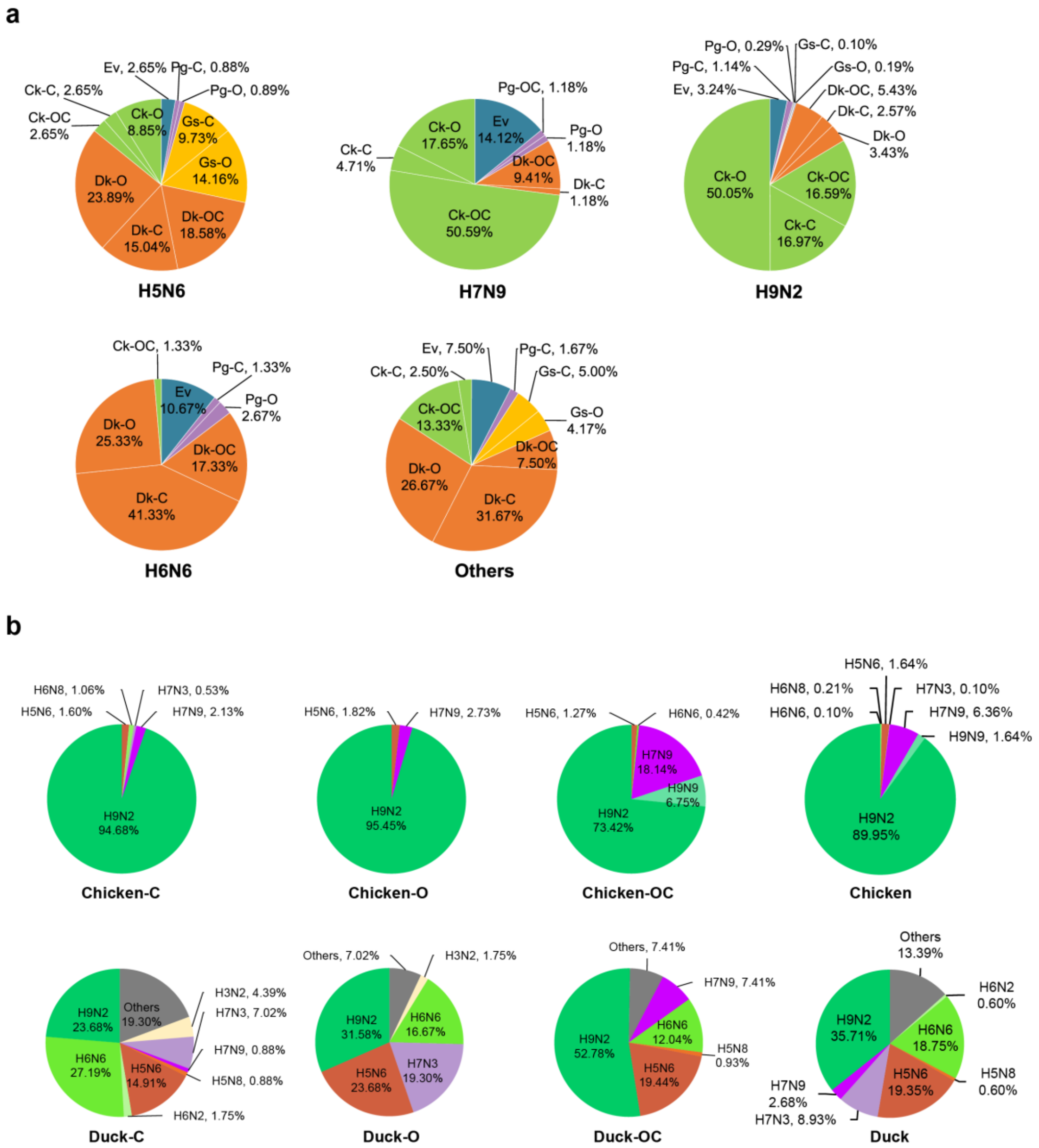

Supplementary Figure 2. Proportion of the pure AIVs isolated from oropharyngeal and/or cloacal swabs. (**a**) Sample types and host species for H5N6, H7N9, H9N2, H6N6, and other AIV subtypes. (**b**) Virus subtype proportions isolated from oropharyngeal and/or cloacal swabs from chickens and ducks. Ck, chicken; Dk, duck; Ev, environment; Gs, goose; Pg, pigeon; -C, cloacal swabs; -O, oropharyngeal swabs; -OC, oropharyngeal and cloacal mixed swabs. Source data are provided as a

Source Data file.

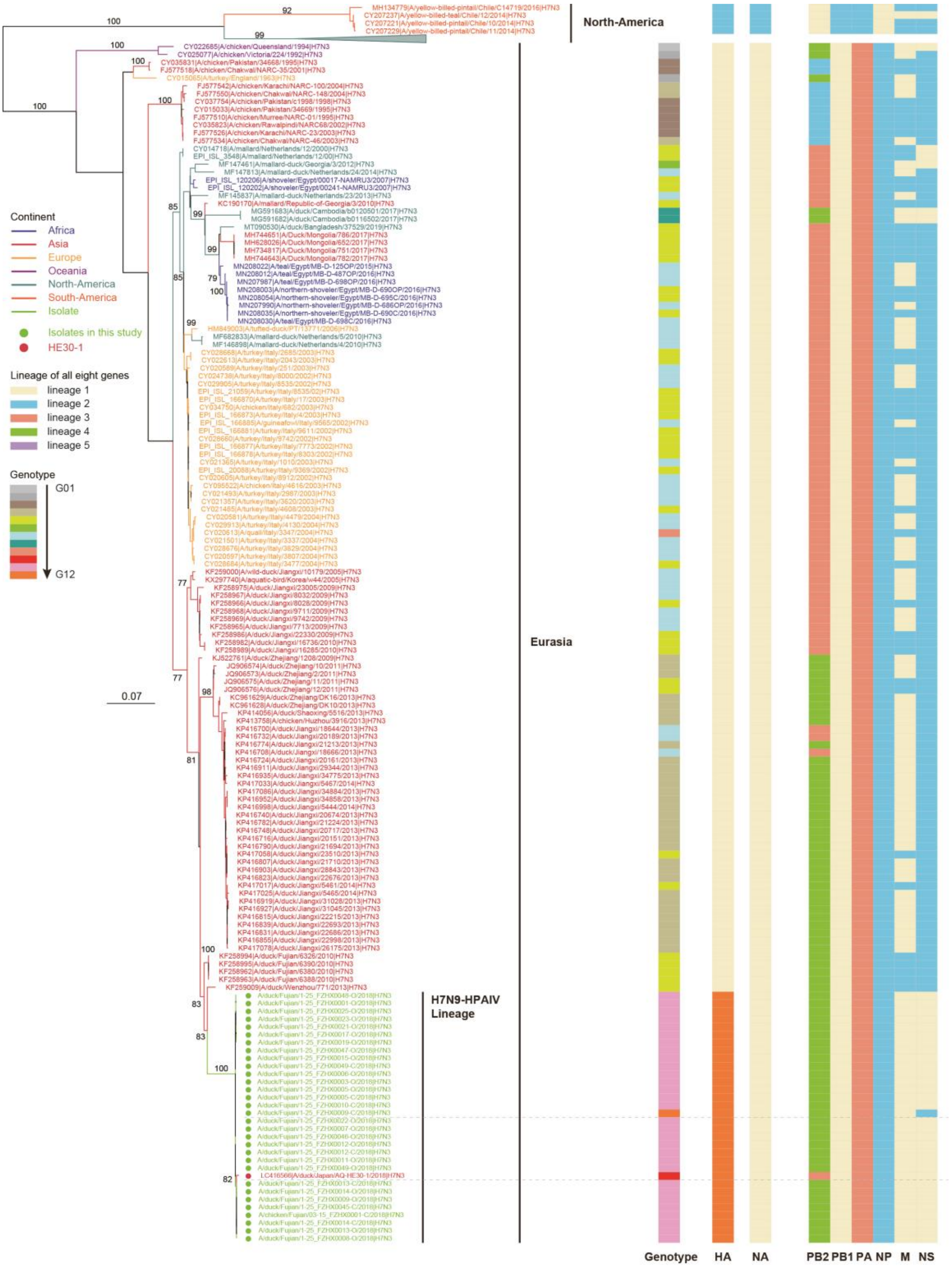

Supplementary Figure 3. Phylogenetic analysis and genotype classification of 615 H7N3 AIVs. In the HA phylogenetic tree of all H7N3 AIVs, the 31 whole-gene H7N3 isolates described in this study are colored in green and highlighted with circles. The

Japanese strain reported by Shibata et al.[1], A/duck/Japan/AQ-HE30-1/2018(H7N3), is highlighted with a red circle. The reference strains with different colors represented strains isolated from Africa, Asia, Europe, Oceania, North-America, South-America, respectively, which were retrieved from the Influenza Virus Resource at National Center for Biotechnology Information (NCBI) and the GISAID database. The ML trees of all the eight genes and the detailed information about genotype classification are provided in Supplementary Data 9 and 10.

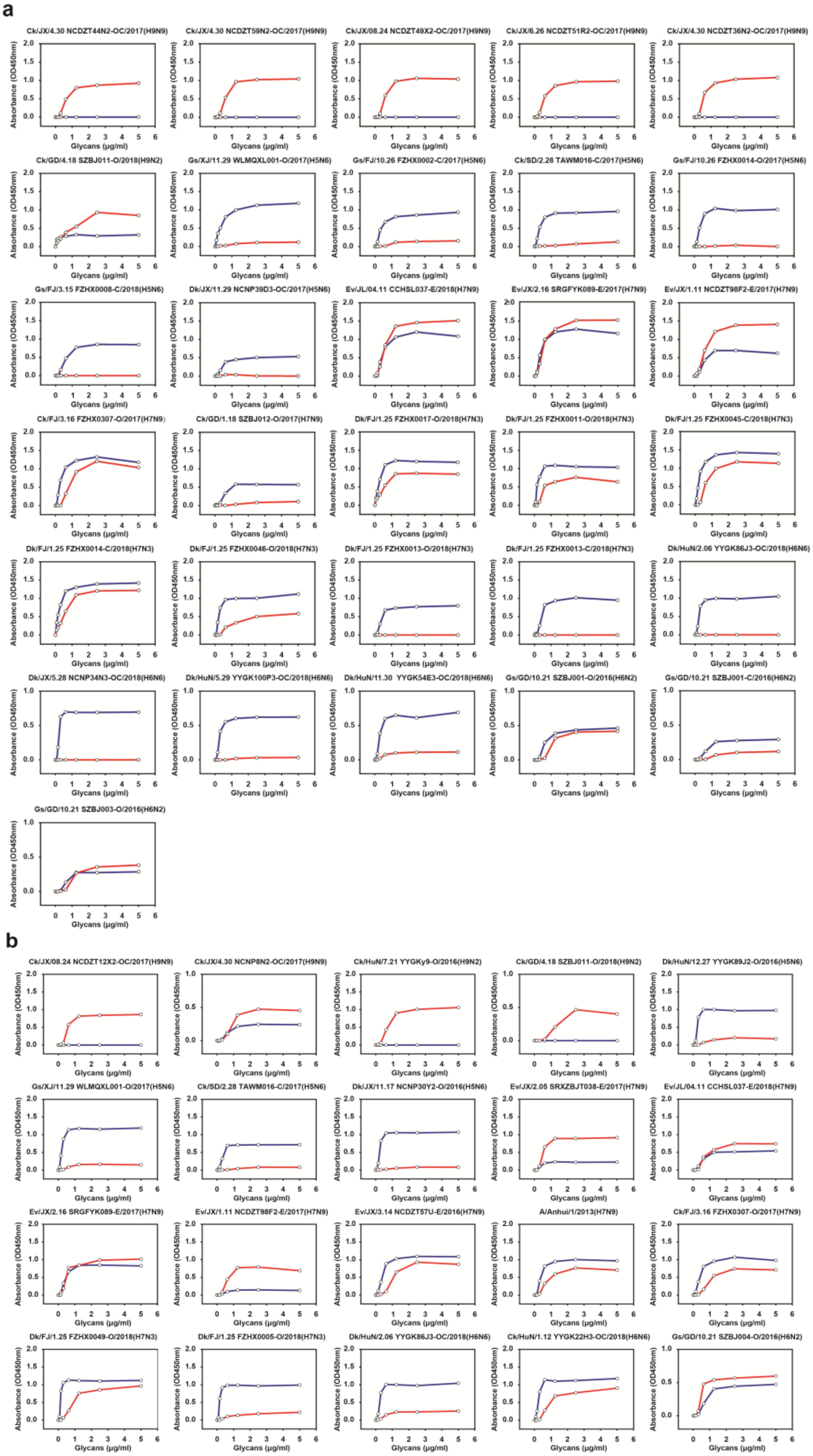

Supplementary Figure 4. Receptor binding properties of additional AIV isolates.

Receptor binding properties of additional AIV isolates described in this study to

human (α2-6-SA) or avian (α2-3-SA) receptors were tested using the solid-phase direct binding assay with trisaccharide (**a**) and pentasaccharide (**b**) receptors. Red and blue lines represent human- and avian-origin receptors, respectively. Source data are provided as a Source Data file.

Supplementary References

1. Shibata, A. et al. Characterization of a novel reassortant H7N3 highly pathogenic avian influenza virus isolated from a poultry meat product taken on a passenger flight to Japan. *J. Vet. Medl. Sci.* **81**, 444-448 (2019).
