# Supplementary material for: Dominant subtype switch in avian influenza viruses during 2016–2019 in China
Source: Nat Commun. 2020 Nov 20;11:5909. doi: 10.1038/s41467-020-19671-3 (PMC7679419; doi:10.1038/s41467-020-19671-3)
Supplement: Supplementary file 2 — Description of Additional Supplementary Files [file 41467_2020_19671_MOESM2_ESM.docx]

**Description of Additional Supplementary Files**

**File Name:** Supplementary Data 1

**Description:** Surveillance of apparently healthy chickens in live poultry markets

**File Name:** Supplementary Data 2

**Description:** Surveillance of apparently healthy ducks in live poultry markets

**File Name:** Supplementary Data 3

**Description:** Surveillance of environmental samples in live poultry markets

**File Name:** Supplementary Data 4

**Description:** Surveillance of apparently healthy pigeons in live poultry markets

**File Name:** Supplementary Data 5

**Description:** Surveillance of apparently healthy geese in live poultry markets

**File Name:** Supplementary Data 6

**Description:** The nucleotide distance matrix between groups of H9 HA, H9 NA, H5 HA, and H7 HA genes

**File Name:** Supplementary Data 7

**Description:** Specific amino acid substitutions in H9, H5, H7, and H6 AIVs

**File Name:** Supplementary Data 8

**Description:** Receptor binding sites on the HA protein and receptor binding properties of H9, H5, H7, and H6 AIVs

**File Name:** Supplementary Data 9

**Description:** The ML trees of HA, NA, PB2, PB1, PA, NP, M, and NS genes of 615 H7N3 AIVs

**File Name:** Supplementary Data 10

**Description:** Detailed information of all the H7N3 viruses analyzed in this study

**File Name:** Supplementary Data 11

**Description:** Detailed information of all primer or probes used in the study
