# Supplementary material for: Dominant subtype switch in avian influenza viruses during 2016–2019 in China
Source: Nat Commun. 2020 Nov 20;11:5909. doi: 10.1038/s41467-020-19671-3 (PMC7679419; doi:10.1038/s41467-020-19671-3)
Supplement: Supplementary file 11 — Supplementary Data 9 [file 41467_2020_19671_MOESM11_ESM.pdf]

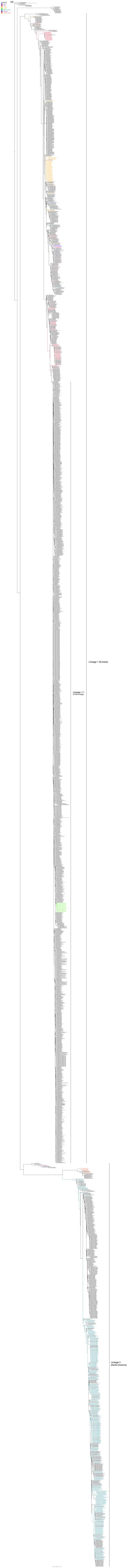
HA
Continent
Africa
Asia
Europe
Isolate
North-America
Oceania
South-America
Lineage 1 (Eurasia)
Lineage 1.1
(H7N9-Lineage)
Lineage 2
(North America)
0.08

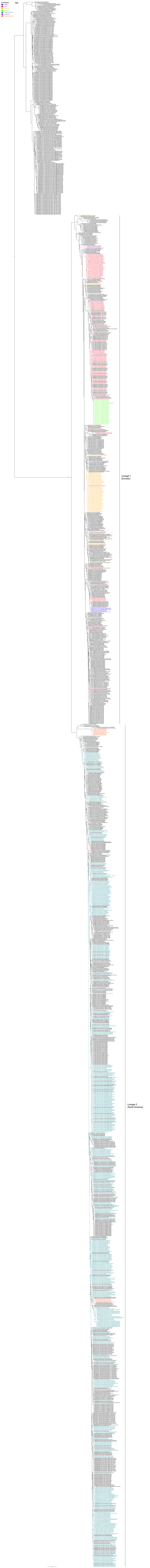
Continent
Africa
Asia
Europe
Isolate
North-America
Oceania
South-America
NA
Lineage 1
(Eurasia)
Lineage 2
(North America)

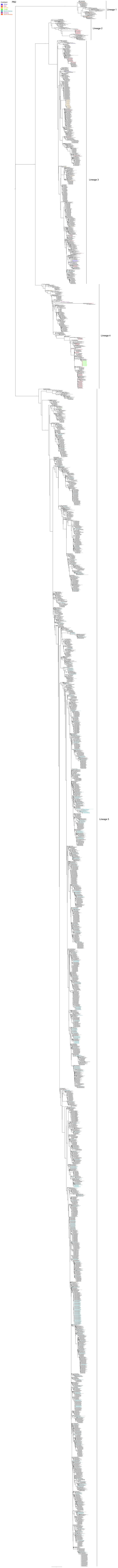

Continent
Africa
Asia
Europe
Isolate
North-America
Oceania
South-America
PB2
Lineage 1
Lineage 2
Lineage 3
Lineage 4
Lineage 5

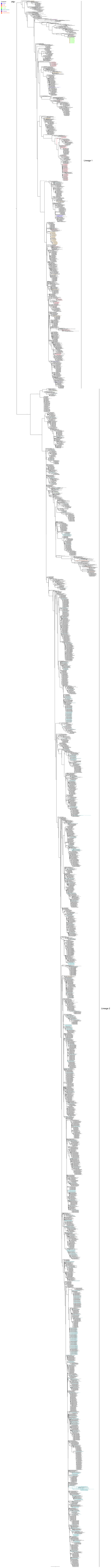
Continent
Africa
Asia
Europe
Isolate
North-America
Oceania
South-America
PB1
Lineage 1
Lineage 2

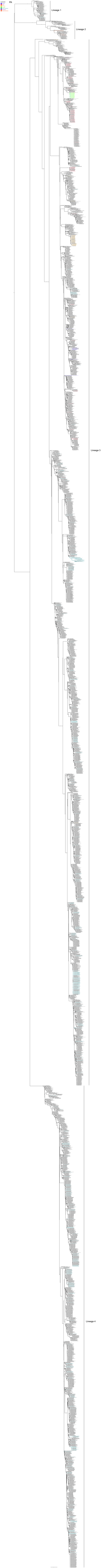
PA
Continent
Africa
Asia
Europe
Isolate
North-America
Oceania
South-America
Lineage 1
Lineage 2
Lineage 3
Lineage 4

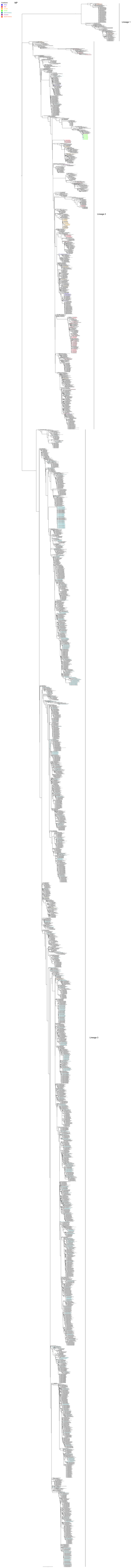

Continent
Africa
Asia
Europe
Isolate
North-America
Oceania
South-America
NP
Lineage 1
Lineage 2
Lineage 3

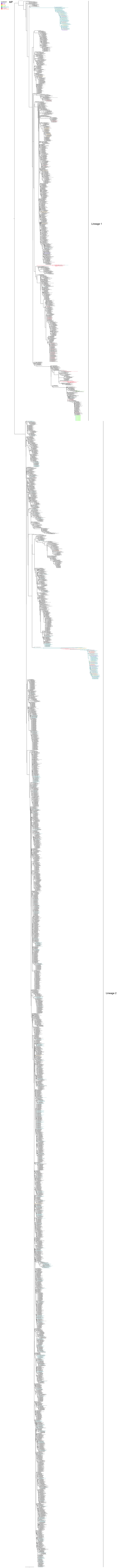
Continent
Africa
Asia
Europe
Isolate
North-America
Oceania
South-America
MP
Lineage 1
Lineage 2

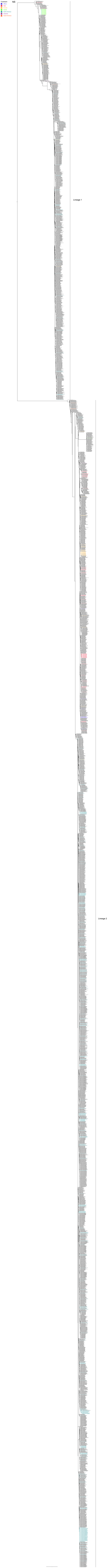
NS
Continent
Africa
Asia
Europe
Isolate
North-America
Oceania
South-America
Lineage 1
Lineage 2
